# Educating on professional habits: attitudes of medical students towards diverse strategies for promoting influenza vaccination and factors associated with the intention to get vaccinated

**DOI:** 10.1186/1472-6920-13-99

**Published:** 2013-07-18

**Authors:** Guillermo Mena, Anna Llupià, Alberto L García-Basteiro, Victor-Guillermo Sequera, Marta Aldea, José María Bayas, Antoni Trilla

**Affiliations:** 1 Preventive Medicine and Epidemiology Unit, Hospital Clinic, c/Rosselló 138, Baixos, Barcelona 08036, Spain; 2 Barcelona Centre for International Health Research (CRESIB, Hospital Clínic-Universitat de Barcelona), c/Rosselló 132, Barcelona 08036, Spain

**Keywords:** Medical students, Vaccination, Influenza, Web 2.0, Strategies

## Abstract

**Background:**

Influenza vaccination coverage in medical students is usually low. Unlike health care workers, there is little information on the attitudes to and predictors of vaccination among medical students, and their attitudes towards institutional strategies for improving rates are unknown.

**Methods:**

This cross-sectional study evaluated the effect of three influenza vaccination promotional strategies (Web page, video and tri-fold brochure) on medical students’ intention to get vaccinated and associated factors. A total of 538 medical students were asked to answer an anonymous questionnaire assessing the intention to get vaccinated after exposure to any of the promotional strategies. Sociodemographic data collected included: sex, age, university year, influenza risk group and cohabiting with member of a risk group.

**Results:**

Four hundred twenty-one students answered the questionnaire, of whom 312 (74.1%) were female, 113 (26.8%) had done clinical rotations, and 111 (26.6%) reported intention to get the flu shot. Logistic regression showed the web group had a greater intention to get vaccinated than the reference group (OR: 2.42 95% CI: 1.16-5.03). Having done clinical rotations (OR: 2.55 95% CI: 1.36-4.38) and having received the shot in previous flu seasons (OR: 13.69 95% CI: 7.86-23.96) were independently associated with the intention to get vaccinated.

**Conclusion:**

Given that previous vaccination is a factor associated with the intention to get vaccinated, education on vaccination of health care workers should begin while they are students, thereby potentiating the habit. In addition, the intention to get vaccinated was greater during the clinical phase of the university career, suggesting this is a good time to introduce promotion strategies. Online promotional campaigns, such as a thematic Web to promote vaccination of health workers, could improve the intention to get vaccinated.

## Background

Hospital outbreaksof influenza have been reported for many decades [1]. The influenza vaccine is safe, and moderately effective in preventing cases of laboratory-confirmed influenza in healthy adults [2]. Influenza transmission is common in health centers, and health care workers (HCW) can act as a vector, transmitting acquired infections to patients. Although leading health organizations recommend influenza vaccination of HCW [3-5],data shows that many remain reluctant to be vaccinated [6].While vaccination is also recommended in medical students doing internships in health centers, coverage in this group tends to be low [7,8], as in HCW, although reports show that college students have a high prevalence of colds and influenza-like illness [9]. Unlike HCW, there is little information on the attitudes to and predictors of vaccination in medical students [10,11], and their attitude to institutional strategies for improving rates is unknown.

The objective of this study was to evaluate the effect of three vaccination promotion strategies on the intention of medical students to get vaccinated and to analyze associated factors.

## Methods

### Settings and Participants

During the 2010/11college year, 979 students (six-year academic course) were enrolled in the Clínic Campus, Faculty of Medicine,University of Barcelona. Students attending classes during the first week of October 2010 (n=538) were invited to participate in this study.

### Study design

We carried out a cross-sectional study that compared the effect of three interventions, representing different influenza vaccination promotion strategies, on the intention to get vaccinated. We included a reference group (control group) who received no intervention.

In our faculty, each of the 6 years of medical studies is allocated to two classrooms. We assigned students to three blocks according to the year (first block: first and second years, second block: third and fourth years, and third block: fifth and sixth years). Thus, each block of two years occupied four classrooms. Each of the four interventions studied was randomly assigned to one classroom in each block, meaning that each intervention was studied in each of the three blocks. Post-intervention, students answered an anonymous questionnaire assessing the intention to be vaccinated.

### Influenza Vaccination Campaign 2010/11

This study was conducted using then-unpublished promotional materials designed by the Preventive Medicine and Epidemiology and Occupational Risk Prevention Units [12].

### Interventions

1. Tri-fold brochure: Containing educational messages and information on risk groups. The brochure was delivered in the classroom and students were asked to read the contents for 10minutes.

2. Video: Presenting the information contained in the brochure and a short film featuring hospital HCW. The video, which lasted about 10minutes, was shown to students in the classroom.

3. Web: Surfing the Web of the 2010/11 influenza campaign, and making use of both the technical information and the games and videos posted on the Web. Students were asked to attend the computer room, where they were provided with a computer and the address of the Web page, which they were asked to navigate for 10minutes.

4. Reference group: No strategy was applied in this group, who solely completed the questionnaire.

A researcher was present in the classroom during all the interventions to ensure that students paid attention to the intervention and that the questionnaire was completed. The researcher also ensured that students only looked at the designated web page during the 10 minutes allotted.

### Data collection and variables

Sociodemographic data collected included sex, age, university year, belonging to influenza risk group and cohabiting with someone belonging to a risk group. Participants were asked about their intention to get vaccinated in the current influenza season and whether they had been vaccinated in any of the past three seasons (’07, ’08 or ’09 -including both seasonal and pandemic H1N1-). Although clinical rotations begin in the fourth year, as the study was carried out at the beginning of the university year, only students in the fifth and sixth years were considered to have done clinical rotations.

### Statistical Analysis

The χ2 test was used to analyze differences in sociodemographic characteristics and variables related to influenza vaccination according to the intervention group. Binary logistic regression was used to identify independent variables related to the main outcome variable intention to get vaccinated. Predictive variables with some association with vaccination (p<0.05) were tested in multivariate models using forward stepwise multivariable logistic regression and the likelihood ratio method. The probability of a type I error in the final models was established as 0.05 (two-tailed). Goodness of fit for the logistic-regression models was examined using the Hosmer-Lemeshow goodness-of-fit test. Data collected were analyzed using SPSS v17.0.

### Ethics

All students were informed orally of the objectives and characteristics of the study and that the questionnaire was anonymous and participation was voluntary. The study was approved by the Hospital Clinic Research Ethics Committee.

## Results

Of the 421 students who answered the questionnaire (participation rate: 78.3%), 312 were female (74.1%), 113 (26.8%) had previously done clinical rotations, 29 (7.0%) reported belonging to risk group for influenza, 80 (19.2%) reported≥1 cohabitants belonged to≥1 risk group and 122(29.0%) reported having received influenza vaccination during at least one of the last three seasons (Table 1). There were no significant differences in sociodemographic characteristics according to the intervention received, except in the Web group, which had a lower proportion of females (60.3%, p=0.007).

One hundred eleven students (26.6%) reported they intended to get vaccinated. Results from the logistic regression model, in Table 2, show that the Web group had a greater intention to get vaccinated than the reference group (OR: 2.42, 95% CI: 1.16-5.03). There was a lower intention to get vaccinated in the tri-fold brochure group (OR: 0.47, 95% CI: 0.23-0.96). Having done clinical rotations (OR: 2.55, 95% CI: 1.36-4.38) and vaccination in previous seasons (OR: 13.69, 95% CI: 7.86-23.96) were independently associated with the intention to get vaccinated.

**Table 1:** Descriptive analysis of student characteristics and the response to questions on students’ role in vaccination promotion according to intervention group

	**Total**	**Control**	**Web**	**Video**	**Tri-fold**	**p**
		**group**			**brochure**	
**Students surveyed**, n	421	128	78	105	110	
**Previous clinical rotations**,	113/421	38/128	12/78	27/105	36/110	
n / total per group (%)	(26.8)	(29.7)	(15.4)	(25.7)	(32.7)	0.051
**Female sex**,	312/421	105/128	47/78	79/105	81/110	
n / total per group(%)	(74.1)	(82.0)	(60.3)	(75.2)	(73.6)	0.007
Students with risk factors*,	29/415	9/128	6/77	5/102	9/108	
n /total per group (%)	(7.0)	(7.0)	(7.8)	(4.9)	(8.3)	0.641
Students cohabiting with total persons with risk factors*,	80/416	29/125	14/78	16/103	21/108	
n / total per group (%)	(19.2)	(23.0)	(17.9)	(15.2)	(19.1)	0.464

**Vaccinated in previous fluseasons**^,	122/421	29/128	22/78	33/105	38/110	
n / total per group (%)	(29.0)	(22.7)	(28.2)	(31.4)	(34.6)	0.215


*Risk factors: Obesity, pregnancy, chronic diseases (respiratory, heart, kidney or liver diseases), neuro or neuromuscular pathologies, spleen disorders, weakened immune system due to HIV infection or treatment that suppresses the immune system, age≥60years, and long-term aspirin therapy.

^Vaccinated in previous flu seasons: Vaccinated in ’07, ’08 or 09’ (including either seasonal or pandemic H1N1) seasons.

p=Differences according to intervention received were calculated using the Chi-square test.

**Table 2:** Factors predictive of the intention to get vaccinated in the bivariate and multivariate analyses (Number of students declaring positive or negative intention=417)

**Characteristic**	**Intention to**	**Bivariate OR (95% CI)**	**P**	**Multivariate OR (95% CI)**	**P**
	**get**				
	**vaccinated**				
**General**	111 / 417				
	(26.6%)				
**Intervention group**			0.028		0.001
Reference	32 (25.4%)	-		-	
Web	31 (39.7%)	1.94 (1.06-3.55)	0.032	2.42 (1.16-5.03)	0.018
Video	26 (24.8%)	0.97 (0.53-1.76)	0.912	0.69 (0.33-1.41)	0.306
Tri-fold brochure	22 (20.4%)	0.75 (0.41-1.39)	0.364	0.47 (0.23-0.96)	0.039
**Previous clinical rotations**					
No	72 (23.6%)	-		-	
Yes	39 (34.8%)	1.73 (1.08-2.77)	0.022	2.55 (1.36-4.38)	0.03
**Sex**					
Male	28 (25.7%)	-			
Female	83 (26.9%)	1.07 (0.65-1.76)	0.798		
Risk factor*					
No	103 (26.4%)	-			
Yes	8 (29.6%)	1.17 (0.50-2.76)	0.715		
**Cohabitant**					
**with person with risk**					
Factor*					
No	85 (25.5%)	-			
Yes	26 (32.9%)	1.03(0.94-1.12)	0.487		
**Vaccinated in**					
**previous flu seasons^**					
No	38 (12.8%)	-		-	
Yes	73 (60.8%)	10.59 (6.42-17.46)	<0.001	13.69 (7.86-23.96)	<0.001

*Risk factors: Obesity, pregnancy, chronic diseases (respiratory, heart, kidney or liver diseases), neuro- or neuromuscular pathologies, spleen disorders, weakened immune system due to HIV infection or treatment that suppresses the immune system, age≥60years, and long-term aspirin therapy.

^Vaccinated in previous flu seasons: Vaccinated in ’07, ’08 or 09’ (including either seasonal or pandemic H1N1) seasons.

The Nagelkerke R Square was 0.364; the Cox and Snell R Square, 0.250; and the Hosmer-Lemeshow goodness-of-fit statistic presented a chi-square of 0.820 (degree of freedom=7, p=0.800), suggesting that the multivariate model was a good fit for the data.

## Discussion

Medical students who surfed the Web 2.0 vaccination page had a greater intention to get vaccinated compared with the reference group who received no intervention. The intention to get vaccinated was greater in students doing clinical rotations and those who had received influenza vaccination in previous seasons.

Investing time and effort in a Web-based strategy seemed to have a greater impact in terms of vaccination promotion in a group of medical students. A similar approach was taken at the University of Wisconsin [13], where researchers designed a web-based tool that allowed medical students to create their own health plan, focused on various factors such as nutrition or choice of lifestyle. The authors concluded that this tool encouraged self-reflection, positive lifestyle habits and education in key aspects of health and well-being. Results supporting web-based promotional strategies were also found in a study in which university students were randomly assigned to receive a web-based intervention involving seven theory-based lessons or to a control group that received minimal information on physical activity. The results showed that the web-based strategy increased the number of days on which students carried out moderate or vigorous physical activity compared with controls and demonstrated that a web-based strategy with attractive contents could induce behavioral changes, supporting the hypothesis of our study [14].

Using a pre-post survey methodology, researchers from Canada compared the impact of two of the most popular YouTube videos discussing and critizingseasonal influenza vaccineon the attitudes towards vaccination of first year medical students. Overall, there were no significant differences in pre and post attitudes to influenza vaccination after viewing the two videos.This suggests that medical students are relatively resistant to the predominately inaccurate, vaccine-critical messaging on YouTube, even when the message is framed as scientific reasoning [15]. In our study, the use of a video including a short film featuring Hospital Clinic HCW and promotional messages apparently had no affect on students’ intention to get vaccinated. Unlike the vaccine-critical content of the videos used in the Canadian research, ours was presented in a friendly format, including four short films on vaccine promotion featuring HCW from the students’ own environment. The video may have had other, unmeasured, effects on the students, but certainly not the one we were looking for.

Our results showed that the tri-fold brochure seemed to have a negative impact, with a smaller number of students in this group reporting an intention to get vaccinated than in the reference group. We have found no reports evaluating the strategy of delivering a similar brochure as an independent intervention. Therefore, it is difficult to determine whether our results were an isolated phenomenon or whether leaflets and brochures are, in fact, ineffective in promoting vaccination.

The web-based strategy was, unlike the other interventions, Interactive. Therefore, future studies could examine whether Interactive offline promotion strategies might have a positive effect on acceptance of influenza vaccination, or whether the strategy needs to be both Interactive and online.

As found in a previous study [11], students who had done clinical rotations were more willing to receive vaccination than the rest. Contact with patients and HCW may have affected student’s attitudes in this respect. The hypothetical coverage that would be achieved in students with clinical experience was not significantly different to that obtained in Hospital Clinic HCW [16], reinforcing the idea that students doing clinical rotations behave more like HCW in their attitude to vaccination. As in HCW, a greater intention to get vaccinated was found in persons vaccinated in previous seasons [17]. This, together with the previously-stated findings, suggests that strategies should be considered for students with clinical experience to ensure that they are vaccinated during their first contacts with patients and that, hopefully, this habit would continue during their professional career.

This study has some limitations. First, we did not design a cluster trail, with the corresponding calculation of sample size. The analytic design, without randomization, produced an imbalance in the number of students included in each group. The difference was greater in the Web group, which was smaller because some students did not agree to attend the computer room for the intervention, resulting in a lower proportion of female students in this group. Nevertheless, given that the variable “sex” was not significantly associated with the intention to get vaccinated in the bivariate analysis forany of the four groups, we did not consider this imbalance as a limiting factor in the regression model. Another limitation was the lack of records with information on whether, during the study season, the student was vaccinated or not after the intervention. While the intention to get vaccinated is not the same as actually being vaccinated, this outcome is widely reported as a proxy in the absence of recorded vaccination [17-19]. The third limitation was the lack of information on the characteristics of students who did not participate in the study. This limits the generalizability of the findings. Another limitation was that the intention to get vaccinated may be conditioned by variables other than those included in the study and which could act as confounders. Lastly, due to the design of this quasi-experimental study and the lack of randomization, we cannot make causal inferences. Therefore, the best option would be to conduct a controlled trial after this first approach to the issue.

## Conclusion

In conclusion, our results suggest that, given that previous vaccination is a factor associated with the intention to get vaccinated, vaccination of physicians should commence in students, thereby potentiating the habit. In addition, the intention to get vaccinated is greater during the clinical phase of the university career, suggesting this is a good time to introduce promotion strategies to strengthen this attitude. The introduction of online interactive promotional campaigns, such as creating a thematic Web for the promotion of vaccination among HCW, could achieve better results in students in terms of intention to get vaccinated than other more-conventional strategies, such as the preparation of brochures or video presentations.

This study formed a part of the larger INTENVAC study of medical students and their attitudes to different tools promoting influenza vaccination and the role of social networks [20,21].

## Competing interests

The authors declare that they have no competing interests. The authors alone are responsible for the content and writing of this article.

## Authors’ contributions

The original idea for the study and primary design of it was carried out by GM. Data were collected by GM, AL, MA, VGS and AT. The review, data analysis and drafting of the manuscript were carried out by GM and AL. ALGB, MA, VGS, JMB and AT helped in the collation and analysis of the information and in the preparation of the manuscript. All authors have approved the final manuscript.

## Pre-publication history

The pre-publication history for this paper can be accessed here:

http://www.biomedcentral.com/1472-6920/13/99/prepub
